# Identification of pyrimethamine- and chloroquine-resistant *Plasmodium falciparum *in Africa between 1984 and 1998: genotyping of archive blood samples

**DOI:** 10.1186/1475-2875-10-388

**Published:** 2011-12-31

**Authors:** Yumiko Saito-Nakano, Kazuyuki Tanabe, Toshihiro Mita

**Affiliations:** 1 Department of Parasitology, National Institute of Infectious Diseases, Shinjuku-ku, Tokyo, Japan; 2 Laboratory of Malariology, Research Institute for Microbial Diseases, Osaka University, Suita, Osaka, Japan; 3 Department of International Affairs and Tropical Medicine, Tokyo Women's Medical University School of Medicine, Shinjuku-ku, Tokyo, Japan

**Keywords:** *Plasmodium falciparum*, Drug resistance, Chloroquine, *pfcrt*, Pyrimethamine, *dhfr*, Africa, Archive sample

## Abstract

**Background:**

Understanding the geographical distribution of drug resistance of *Plasmodium falciparum *is important for the effective treatment of malaria. Drug resistance has previously been inferred mainly from records of clinical resistance. However, clinical resistance is not always consistent with the parasite's genetic resistance. Thus, molecular identification of the parasite's drug resistance is required. In Africa, clinical resistance to pyrimethamine (Pyr) and chloroquine (CQ) was evident before 1980 but few studies investigating the genetic resistance to these drugs were conducted before the late 1990s. In this study, genotyping of genes involved in resistance to Pyr and CQ was performed using archive blood samples from Africa between 1984 and 1998.

**Methods:**

Parasite DNA was extracted from *P. falciparum*-infected blood smears collected from travellers returning to Japan from Africa between 1984 and 1998. Genotypes of the dihydrofolate reductase gene (*dhfr*) and CQ-resistance transporter gene (*pfcrt) *were determined by polymerase chain reaction amplification and sequencing.

**Results:**

Genotyping of *dhfr *and *pfcrt *was successful in 59 and 80 samples, respectively. One wild-type and seven mutant *dhfr *genotypes were identified. Three *dhfr *genotypes lacking the S108N mutation (NRSI, ICSI, IRSI; amino acids at positions 51, 59, 108, and 164 with mutations underlined) were highly prevalent before 1994 but reduced after 1995, accompanied by an increase in genotypes with the S108N mutation. The *dhfr *IRNI genotype was first identified in Nigeria in 1991 in the present samples, and its frequency gradually increased. However, two double mutants (ICNI and NRNI), the latter of which was exclusively found in West Africa, were more frequent than the IRNI genotype. Only two *pfcrt *genotypes were found, the wild-type and a Southeast Asian type (CVIET; amino acids at positions 72-76 with mutations underlined). The CVIET genotype was already present as early as 1984 in Tanzania and Nigeria, and appeared throughout Africa between 1984 and 1998.

**Conclusions:**

This study is the first to report the molecular identification of Pyr- and CQ-resistant genotypes of *P. falciparum *in Africa before 1990. Genotyping of *dhfr *and *pfcrt *using archive samples has revealed new aspects of the evolutionary history of Pyr- and CQ-resistant parasites in Africa.

## Background

Drug resistance of *Plasmodium falciparum*, the most virulent human malaria parasite, is imposing a serious problem for the effective treatment of malaria in almost all endemic areas. Thus it is imperative to understand the geographical distribution and origin of the parasite's drug resistance. In Africa, clinical resistance of falciparum malaria to chloroquine (CQ) was first reported in the late 1970s [1,2]. In addition, while clinical resistance to pyrimethamine (Pyr) had been reported in limited endemic foci in the 1950s after mass drug treatment using Pyr [3,4], highly resistant parasites became remarkably frequent and spread into endemic regions throughout Africa during the 1990s after the implementation of sulphadoxine/pyrimethamine (SP) as a first-line therapy in these regions [5]. Clinical resistance is not always consistent with genetic resistance because drug resistance results from the interplay of the parasite, drug, and human host, and is largely influenced by immune factors. In highly endemic areas in Africa, where individuals often possess semi-immunity against malaria after repeated infections, this semi-immunity more or less strengthens efficacy of anti-malarial drugs to drug-resistant parasites [6,7]. Thus, in Africa the timing of the first appearance of genetic resistance to CQ and Pyr and its subsequent spread in the continent remain to be explored.

The *P. falciparum *chloroquine-resistance transporter gene (*pfcrt*) was identified as a primary target gene for CQ resistance in 2000 [8]. PfCRT is a transmembrane protein, localized to the parasite's food vacuole [9]. An amino acid substitution at position 76 from lysine to threonine (K76T) in *pfcrt *confers resistance to CQ [8]. Pyr inhibits a key enzyme in the folate biosynthetic pathway, dihydrofolate reductase (DHFR), which is encoded by the parasite dihydrofolate reductase gene (*dhfr*). Four major amino acid substitutions in *dhfr *have been widely recognised among Pyr-resistant parasites [10,11]. A mutation at position 108 from serine to asparagine (S108N), leading to the NCNI *dhfr *genotype (amino acids at positions 51, 59, 108, and 164 with mutation underlined), is essential for conferring a mild resistance to Pyr in the parasite [10-13]. Additional point mutations at 51, 59, and 164 are associated with increased levels of Pyr resistance *in vitro *[11]. Importantly, as the number of mutations in *dhfr *increases, levels of resistance to Pyr become higher [13]. The IRNL* dhfr *genotype of the quadruple mutant shows so far the highest IC_50 _values to Pyr (1,111-fold as compared to that of the wild-type parasite) [13]. The IRNL *dhfr *genotype is predominant in Southeast Asia and South America where the efficacy of SP has already reached unacceptable levels [14].

Recent molecular studies using microsatellite markers showed that the geographical origins of the mutant *pfcrt *genotypes were extremely restricted and resistant genotypes spread into almost all endemic regions, due to a selective sweep driven by treatment with CQ [14-17]. The CVIET (amino acids at positions 72-76 in *pfcrt *with mutations underlined) genotype represents the most common CQ-resistant type in Southeast Asia, which expanded to Africa. Similarly, a highly Pyr-resistant *dhfr *genotype (IRNI triple mutant) emerged from Southeast Asia and migrated to Africa, where it spread into endemic regions throughout Africa [14,18,19].

The identification of *pfcrt *and *dhfr *as target genes for drug resistance provides solid ground for exploring the geographical distribution and spread of CQ- and Pyr-resistant *P. falciparum*. However, previous reports of genetic resistance (but not clinical resistance) to these drugs are very limited before the 1990s, since the target genes for CQ- and Pyr-resistance were not identified until 2000 and 1988, respectively [8,10,20]. Indeed, studies on the genotypes of drug-resistant genes are scarce, with only one report each for *pfcrt *[21] and *dhfr *[22] in the late 1980s in Africa. Investigating the geographical distribution of *pfcrt *and *dhfr *genotypes in Africa before 1990 is undoubtedly crucial for tracing the evolutionary history of CQ- and Pyr-resistance in the continent, where both CQ- and Pyr-resistance was introduced from Southeast Asia. To address this issue, genotyping of archive blood samples was performed using samples collected from travellers who had returned from Africa between 1984 and 1998. Results show that in Africa the CQ-resistant *pfcrt *genotype was already widely prevalent in the 1980s, and the highly Pyr-resistant *dhfr *genotypes (double and triple mutants) increased after the mid-1990s.

## Methods

### Collection of samples and DNA extraction

The archive blood samples used in this study had been collected and stored as blood smears for the national surveillance system of imported malaria cases operating in 50 hospitals in Japan from 1984 to 1998 [23]. Of the 588 malaria cases imported to Japan during the period, 122 *Plasmodium falciparum *cases were derived from 23 African countries. Parasite DNA was extracted from Giemsa-stained thin blood smears as previously described [23]. Briefly, Giemsa-stained slides were dipped in xylene and then in methanol to remove the immersion oil and dye, respectively. Each blood smear was scraped off with the edge of a clean glass slide, and subjected to DNA purification using the QIAamp DNA Blood Mini Kit (Qiagen, Hilden, Germany). A non-infected blood smear was also used as a negative control in the DNA extraction and polymerase chain reaction (PCR) amplification procedures. In total, genotyping was successful for *dhfr *and/or *pfcrt *in 85 samples of the 122 samples examined [see Additional file 1].

### Molecular analysis

For genotyping of the *dhfr *locus, amplification of a 350-base pair fragment covering the four polymorphic sites at positions 51, 59, 108 and 164 was unsuccessful, due primarily to severe fragmentation of the templates extracted from the methanol-fixed blood smears, which were not suitable for amplification of fragments greater than 200-base pairs [24,25]. Thus, three 190-base pair fragments covering the polymorphic amino acid positions at 51 and 59, 108, and 164 were separately amplified by nested PCR [23]. The PCR products were subjected to direct sequencing in both directions. In samples with mixed infections of different genotypes showing superimposed peaks on an electropherogram, the PCR products were cloned into pT7Blue vector (Merck, Darmstadt, Germany) and sequenced. More than 10 plasmid clones were sequenced to ascertain linkage of the polymorphisms at 51 and 59 in *dhfr*; those clones in which linkage was not confirmed were excluded from further analysis. For genotyping of the *pfcrt *locus, a 190-base pair fragment of *pfcrt *encoding the amino acid residues at positions 72-76 was amplified by nested PCR as previously described [5].

### Statistical analysis

Comparisons between the prevalence of drug resistant genotypes before and after 1995 were examined using the Fisher exact test or Chi-square analysis. A *p*-value of less than 0.05 was considered to be statistically significant.

## Results

### Pyrimethamine-resistant genotype

**Figure 1:**
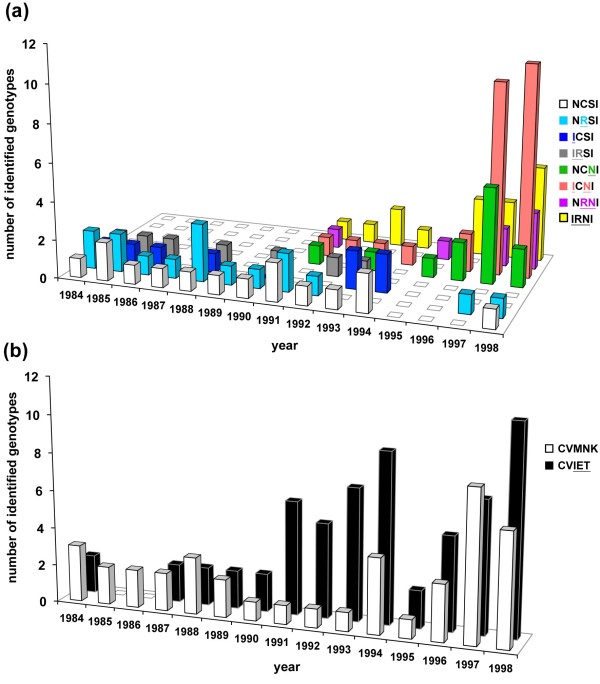
**Yearly change in the number of *dhfr*- and *pfcrt*-genotypes in Africa between 1984 and 1998**. (**a**) Wild-type (NCSI), pyrimethamine-sensitive (NRSI, ICSI, IRSI) and pyrimethamine-resistant (NCNI, ICNI, NRNI, IRNI) *dhfr *genotypes were identified in blood samples collected from cases of *Plasmodium falciparum *malaria in Africa between 1984 and 1998. The height of each coloured bar shows the number of samples in that year infected with that particular genotype. (**b**) Wild-type (CVMNK; white bars) and chloroquine-resistant (CVIET; black bars) *pfcrt *genotypes were identified in blood samples collected as above. The height of each bar shows the number of samples in that year infected with that genotype.

**Figure 2:**
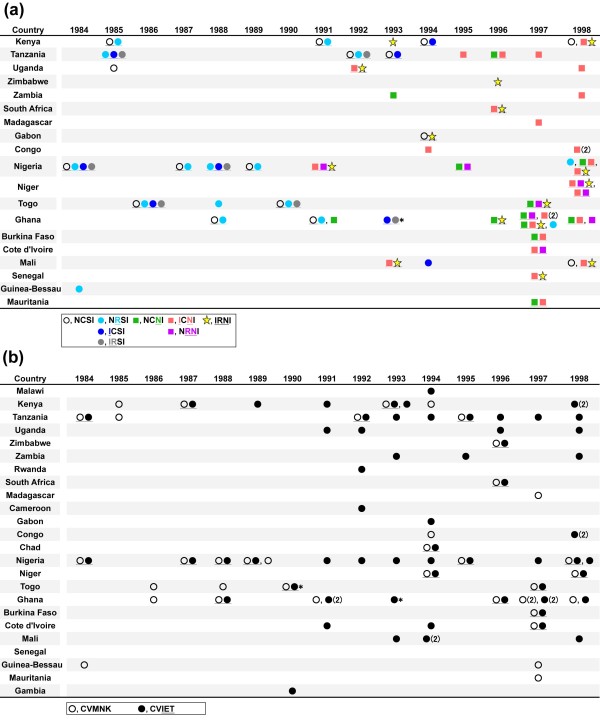
**Time-line scheme for *dhfr *and *pfcrt *genotypes determined using samples from Africa between 1984 and 1998**. (**a**) Wild-type (NCSI), pyrimethamine-sensitive (NRSI, ICSI, IRSI) and pyrimethamine-resistant (NCNI, ICNI, NRNI, IRNI) *dhfr *genotypes of *Plasmodium falciparum *are shown by the different coloured symbols, as listed at the bottom of the figure. In samples with mixed genotype infections, the different genotypes are listed together and underlined. Values in parentheses are the number of samples. *One individual that returned from Ghana in 1993 also visited Burkina Faso in the same trip. (**b**) Wild-type (CVMNK) and chloroquine-resistant (CVIET) *pfcrt *genotypes of *P. falciparum *are shown by white and black circles, respectively. In samples with mixed genotype infections, the different genotypes are listed together and underlined. Values in parentheses are the number of samples. *One individual returning from Ghana in 1993 and one individual returning from Togo in 1990 also visited Burkina Faso in the same trip.

Genotyping of *dhfr *was successful in 59 of the 122 archive samples, of which 37 (63%) showed mixed infections of different *dhfr *genotypes (Figures 1a and 2a). In addition to the wild-type (NCSI), seven mutant genotypes were identified: three with a single mutation (NRSI, ICSI, NCNI), three with double mutations (IRSI, ICNI, NRNI), and one with a triple mutation (IRNI). The most Pyr-resistant genotype (IRNL) known so far was not found in the present study. Of note, the three mutant genotypes lacking the S108N mutation (NRSI, ICSI, IRSI) were more prevalent before 1994 than after 1995 (p < 0.0001, Fisher exact test).

The IRNI genotype, which is currently predominant in many endemic regions in Africa [26], was first identified in Nigeria in 1991 in the present samples (Figure 2a). Since then, the frequency of IRNI genotype gradually increased (Figure 1a), but it was not highly predominant in both East Africa (16%) and West Africa (18%) in 1991-1998. Instead, the double mutant genotypes (ICNI and NRNI) were predominant during that period in the present sample set. The NRNI genotype was identified exclusively in West African countries: Nigeria, Ghana, Cote d'Ivoire, and Niger (Figure 2a). On the other hand, the ICNI genotype was prevalent in both East and West Africa.

### Chloroquine-resistant genotype

Genotyping of *pfcrt *was successful in 80 of the 122 archive samples. Only two *pfcrt *genotypes were found; the wild-type (CVMNK) and the CQ-resistant genotype (CVIET) of Southeast Asia (Figure 1b). Mixed infections of wild-type and CVIET type were found in 22 (28%) samples. The SVMNT* pfcrt *genotype that originated in either Melanesia or South America was not detected. The CVIET genotype was already present as early as 1984 in Tanzania and Nigeria in the present samples (Figure 2b), and appeared throughout the studied period (1984-1998). There was no difference in the prevalence of the CQ-resistant *pfcrt *genotype (CVIET) before 1994 and after 1995 (p > 0.8, Chi-square analysis). The prevalence of CQ-resistant *pfcrt *genotype between West and East Africa was comparable (p > 0.48, Chi-square test).

## Discussion

The present study is the first to report *pfcrt *and *dhfr *genotypes in Africa before 1990 using archive samples. It was found that before the mid-1990s the predominant *dhfr *genotypes were wild-type (NCSI) and three mutant genotypes (NRSI, ICSI, IRSI), all of which lacked the S108N mutation. This is unexpected because it was believed that the initial mutation in *dhfr *was exclusively the S108N mutation, which confers weak resistance to Pyr on *P. falciparum *[10-13]. Experimental investigations have recently demonstrated that mutant genotypes lacking the S108N mutation (NRSI, ICSI, IRSI) are as sensitive to Pyr as the wild-type parasite [13,27]. Notably, the prevalence of these three mutant genotypes (NRSI, ICSI, IRSI) lacking the S108N mutation, as well as the wild-type, was remarkably reduced after the mid-1990s. Instead, *dhfr *mutant genotypes possessing the S108N mutation became more frequent in the late 1990s than in the early 1990s. SP had been used as a secondary treatment for CQ-resistant falciparum malaria in limited African countries before the mid-1990s [5], but was widely deployed in many African countries following the first official introduction of SP as the first-line treatment for uncomplicated malaria in Malawi in 1993. Therefore it seems likely that increased Pyr pressure may have led to the increased prevalence of *dhfr *genotypes with the S108N mutation, and the observed reduction in the three Pyr-sensitive genotypes (NRSI, ICSI, IRSI) and wild-type (NCSI) in Africa.

This study also showed that the *dhfr *triple mutant (IRNI), a highly Pyr-resistant genotype, was already present in Central and West Africa in the early 1990s; Nigeria in 1991, Uganda in 1992, and Mali in 1993. The earliest appearance of the IRNI genotype, known to date, was in 1988 in Kenya [22], and this genotype was shown to have been introduced from Southeast Asia. Most of the IRNI genotype currently prevalent in Africa has been generated from this migrated resistant parasite lineage [14,18,19,28], which arrived in East Africa then rapidly moved westwards. Thus, the identification of the IRNI genotype in Nigeria in 1991 supports the idea that the Southeast Asian resistant parasite had already migrated into Central/West Africa in the early 1990s, during which time treatment with Pyr was not common in Africa. However, this idea needs to be confirmed because migration of this type from Southeast Asia cannot be substantiated without the assessment of microsatellite haplotypes flanking *dhfr *[18,19]. Indeed, an indigenous origin of the same triple mutant (IRNI) genotype, having microsatellite haplotypes distinctive from the Southeast Asian haplotypes, has recently been shown in Cameroon [29] and Kenya [14,30]. In the present study, unfortunately, it was not feasible to determine the lineage of the resistant parasite with the triple mutant (IRNI) genotype, as sufficient amounts of DNA required to determine the microsatellite haplotypes could not be recovered from the Giemsa-stained thin blood smears. However, the recently discovered indigenous African parasite lineage was observed in low frequency compared to the Southeast Asian parasite lineage [14,29]. Hence, it seems likely that the Southeast Asian parasite lineage had already migrated to Africa before SP was widely used in Africa, and subsequently spread across the continent after the increased use of SP.

The highest Pyr-resistant *dhfr *genotype, quadruple mutant (IRNL), was not identified in the present sample set. Consistently, the earliest record of the appearance of IRNL genotype in Africa was in 1999 from Uganda [31]-in 2000 from Tanzania [32]. However, the prevalence of the IRNL genotype is notably still low in many endemic countries in Africa [30] in spite of the intense usage of SP as a first-line therapy [33]. This is in sharp contrast to a high prevalence of the IRNL genotype in Indochina, where the genotype has remarkably increased after the initial identification in the late 1980s [33,34]. Many factors appear to be associated with the observed difference of the prevalence of the IRNL genotype between Africa and Indochina. One factor could be a level of acquired immunity against falciparum malaria. In many endemic areas in Africa, adults acquire semi-immunity after repeated infections. In those individuals SP is considered to be effective to not only Pyr-sensitive parasites but also Pyr-resistant parasites. Importantly, the IRNL genotype is reportedly not associated with SP treatment failure or *in vivo *resistance in individuals who have high levels of immunity to malaria [33,35]. It is thus suggested that the IRNL genotype has not been strongly selected for by SP pressure in semi-immune African adults, and consequently the genotype has not expanded in Africa. Other factors that have retarded the expansion of the IRNL genotype in Africa cannot be excluded, and this issue needs to be clarified in the near future.

This study is the first to report the occurrence of the CVIET CQ-resistant *pfcrt *genotype as early as 1984 in Nigeria. Previously, the oldest identification of the *pfcrt *mutant genotype was in 1989 in East Africa [21] and in 1992 in West Africa [36]. This strongly suggests that CQ resistance, which first arrived in East Africa from Southeast Asia, had already spread to West Africa by 1984. Around that time, however, CQ efficacy was satisfactory in the countries of western Africa, and several *in vivo *tolerance and resistance cases were reported only in non-immune individuals [37-39]. Acquired immunity against malaria in individuals in highly endemic areas in Africa may have strengthened the efficacy of CQ against the CQ-resistant parasites [6,7] and probably masked the appearance of clinical CQ resistance.

In contrast to the mainland of Africa, where resistance to CQ and Pyr is widely spread, CQ and Pyr are still effective in Madagascar and Comoros in the south-western Indian Ocean [40]. A recent report has shown that Pyr resistant genotypes found in Madagascar were introduced from Comoros [41]. Unfortunately, the present sample set included only one sample from Madagascar, and thus it is difficult to infer when and from where drug resistant genotype was introduced to Madagascar and Comoros islands. Further molecular surveys of samples from African countries including Madagascar and Comoros are required for better understanding of the evolutionary history of drug resistance in the continent.

## Conclusions

The present study used archive blood samples to reveal new aspects of the evolutionary history of *Plasmodium falciparum *resistance to Pyr and CQ in Africa. Pyr-sensitive mutant genotypes lacking the S108N mutation were frequently observed in the 1980s but were drastically reduced in the late 1990s, and instead, the frequency of highly Pyr-resistant genotypes (double and triple mutants) considerably increased after the mid-1990s. In addition, the CQ-resistant *pfcrt *genotype, CVIET, was identified as early as 1984 in West Africa. Further molecular epidemiological investigations using archive samples from diverse endemic areas would lead to a better understanding of the evolutionary history of drug resistance of *P. falciparum*.

## Competing interests

The authors declare that they have no competing interests.

## Authors' contributions

YSN conducted the genotyping, data collection, and prepared the manuscript. KT participated in the design of the study, and provided critical input on the manuscript. TM assisted with the population genetic analysis, and contributed helpful input to the manuscript. All authors have read and approved the final version of the manuscript.

## Supplementary Material

Additional file 1 **Table S1. Plasmodium falciparum pfcrt and dhfr genotypes determined in 85 blood smears from Africa between 1984 and 1998**. **Footnotes: **The *pfcrt *and *dhfr *genotypes determined are shown for each individual sample. Mutated amino acid residues are underlined. *pfcrt*, *P. falciparum *chloroquine-resistance transporter; *dhfr*, dihydrofolate reductase; nd, not determined. Click here for file
