# Supplementary material for: Identification of pyrimethamine- and chloroquine-resistant Plasmodium falciparum in Africa between 1984 and 1998: genotyping of archive blood samples
Source: Malar J. 2011 Dec 31;10:388. doi: 10.1186/1475-2875-10-388 (PMC3309963; doi:10.1186/1475-2875-10-388)
Supplement: Additional file 1 — Table S1. Plasmodium falciparum pfcrt and dhfr genotypes determined in 85 blood smears from Africa between 1984 and 1998. Footnotes: The pfcrt and dhfr genotypes determined are shown for each individual sample. Mutated amino acid residues are underlined. pfcrt, P. falciparum chloroquine-resistance transporter; dhfr, dihydrofolate reductase; nd, not determined. [file 1475-2875-10-388-S1.PDF]

Supplementaly Table 1. *Plasmodium falciparum pfcrt* and *dhfr* genotypes determined in 85 blood smears from Africa between 1984 and 1998

Sample Number	Year	Country	*Pfcrt* Genotypes		*Pfdhfr* Genotypes							
1	1984	Tanzania	CVMNK	CV**IET**				nd				
2		Nigeria	CVMNK	CV**IET**	NCSI	N**R**SI	**I**CSI	**IR**SI				
3		Guinea-Bessau	CVMNK			N**R**SI						
4	1985	Kenya	CVMNK		NCSI	N**R**SI						
5		Tanzania	CVMNK			N**R**SI	**I**CSI	**IR**SI				
6		Uganda	nd		NCSI							
7	1986	Togo	CVMNK		NCSI	N**R**SI	**I**CSI	**IR**SI				
8		Ghana	CVMNK					nd				
9	1987	Kenya	CVMNK	CV**IET**				nd				
10		Nigeria	CVMNK	CV**IET**	NCSI	N**R**SI						
11	1988	Nigeria	CVMNK	CV**IET**		N**R**SI	**I**CSI	**IR**SI				
12		Togo	CVMNK			N**R**SI						
13		Ghana	CVMNK	CV**IET**	NCSI	N**R**SI						
14	1989	Kenya		CV**IET**				nd				
15		Nigeria	CVMNK					nd				
16		Nigeria	CVMNK	CV**IET**	NCSI	N**R**SI						
17	1990	Togo, Burkina Faso	CVMNK	CV**IET**	NCSI	N**R**SI		**IR**SI				
18		Gambia		CV**IET**				nd				
19	1991	Kenya		CV**IET**	NCSI	N**R**SI						
20		Uganda		CV**IET**				nd				
21		Nigeria		CV**IET**						**I**C**N**I	N**RN**I	**IRN**I
22		Ghana	CVMNK						NC**N**I			
23		Ghana		CV**IET**				nd				
24		Ghana		CV**IET**	NCSI	N**R**SI						
25		Cote d'Ivoire		CV**IET**				nd				
26	1992	Tanzania	CVMNK	CV**IET**	NCSI	N**R**SI	**I**CSI					
27		Uganda		CV**IET**						**I**C**N**I		**IRN**I
28		Rwanda		CV**IET**				nd				
29		Cameroon		CV**IET**				nd				
30		Nigeria		CV**IET**				nd				
31	1993	Kenya		CV**IET**								**IRN**I
32		Kenya	CVMNK	CV**IET**				nd				
33		Tanzania		CV**IET**	NCSI		**I**CSI					
34		Zambia		CV**IET**					NC**N**I			
35		Nigeria		CV**IET**				nd				
36		Ghana, Burkina Faso		CV**IET**		N**R**SI	**I**CSI					
37		Mali		CV**IET**						**I**C**N**I		**IRN**I
38	1994	Malawi		CV**IET**				nd				
39		Kenya	CVMNK		NCSI		**I**CSI					
40		Tanzania		CV**IET**				nd				
41		Gabon		CV**IET**	NCSI							**IRN**I
42		Congo	CVMNK							**I**C**N**I		
43		Chad	CVMNK	CV**IET**				nd				
44		Nigeria		CV**IET**				nd				
45		Niger	CVMNK	CV**IET**				nd				
46		Cote d'Ivoire		CV**IET**				nd				
47		Mali		CV**IET**			**I**CSI					
48		Mali		CV**IET**				nd				
49	1995	Tanzania	CVMNK	CV**IET**				nd				
50		Tanzania	nd							**I**C**N**I		
51		Zambia		CV**IET**				nd				
52		Nigeria	CVMNK	CV**IET**					NC**N**I		N**RN**I	
53	1996	Tanzania		CV**IET**					NC**N**I	**I**C**N**I		
54		Uganda		CV**IET**				nd				
55		Zimbabwe	CVMNK	CV**IET**								**IRN**I
56		South Africa	CVMNK	CV**IET**						**I**C**N**I		**IRN**I
57		Ghana	CVMNK	CV**IET**					NC**N**I			**IRN**I
58	1997	Tanzania		CV**IET**						**I**C**N**I		
59		Madagascar	CVMNK							**I**C**N**I		
60		Nigeria		CV**IET**				nd				
61		Togo	CVMNK	CV**IET**					NC**N**I	**I**C**N**I		**IRN**I
62		Ghana	CVMNK							**I**C**N**I		
63		Ghana	CVMNK						NC**N**I		N**RN**I	
64		Ghana		CV**IET**						**I**C**N**I		
65		Ghana		CV**IET**						**I**C**N**I	N**RN**I	
66		Burkina Faso	CVMNK	CV**IET**					NC**N**I	**I**C**N**I		
67		Cote d'Ivoire	CVMNK	CV**IET**						**I**C**N**I	N**RN**I	
68		Guinea-Bessau	CVMNK							**I**C**N**I		**IRN**I
69		Mauritania	CVMNK						NC**N**I	**I**C**N**I		
70	1998	Kenya		CV**IET**						**I**C**N**I		**IRN**I
71		Kenya		CV**IET**	NCSI							
72		Tanzania		CV**IET**				nd				
73		Uganda		CV**IET**						**I**C**N**I		
74		Zambia		CV**IET**						**I**C**N**I		
75		Congo		CV**IET**						**I**C**N**I		
76		Congo		CV**IET**						**I**C**N**I		
77		Nigeria	nd			N**R**SI						
78		Nigeria		CV**IET**					NC**N**I	**I**C**N**I		
79		Nigeria	CVMNK	CV**IET**						**I**C**N**I		**IRN**I
80		Niger	nd							**I**C**N**I	N**RN**I	**IRN**I
81		Niger	CVMNK	CV**IET**						**I**C**N**I	N**RN**I	
82		Ghana	CVMNK								N**RN**I	
83		Ghana		CV**IET**					NC**N**I	**I**C**N**I		
84		Mali	nd		NCSI							
85		Mali		CV**IET**						**I**C**N**I		**IRN**I
Total (n = 85)			38	64	16	16	9	5	11	28	8	14
